# A prospective, randomized, controlled, double-blind, multi-center study to evaluate the efficacy and safety of a blue light device for the treatment of chronic back pain

**DOI:** 10.3389/fpain.2024.1444401

**Published:** 2024-07-23

**Authors:** Ralf Baron, Bart Morlion, Albert Dahan, Michael Überall, Golo von Basum, Imane Wild

**Affiliations:** ^1^ Division of Neurological Pain Research and Therapy, Department of Neurology, Christian-Albrechts-Universitaet Kiel, Kiel, Germany; ^2^ Leuven Centre for Algology & Pain Management, University Hospitals of Leuven, Leuven, Belgium; ^3^ Department of Anesthesiology, Leiden University Medical Center, Leiden, Netherlands; ^4^ Algesiology and Pediatrics (IFNAP), Institute for Neurosciences, Institute of Neurological Sciences, Nuernberg, Germany; ^5^ Heat2Move BV, Amsterdam, Netherlands

**Keywords:** pain, chronic, low back, phototherapy, blue light

## Abstract

**Introduction:**

Chronic back pain is one of the most prevalent conditions and has a large socio-economic impact. The lack of routine use of non-pharmacological options and issues associated with pharmacological treatments underscore high unmet needs in the treatment of back pain. Although blue light phototherapy has proven efficacy in dermatology, limited information is available about its use in back pain.

**Methods:**

In this proof-of-concept, randomized controlled trial, a pain relief patch (PRP) delivered blue light at the site of back pain for 30 min during five treatment sessions. The comparator device delivered green light for 5 s but was worn for 30 min. A follow-up visit took place after the last treatment. The primary objective was to demonstrate the superiority of treatment by PRP, compared to the control device, in reducing pain intensity at the end of the treatment period. The post-treatment visual analog scale (VAS) pain intensity score for each group was calculated across the five treatment sessions and compared to the baseline. Secondary objectives included the disability score (Roland–Morris Disability Questionnaire) and safety.

**Results:**

The full analysis set included 171 patients. A statistically significant reduction in pain intensity occurred after the use of PRP (*p* < 0.02), but the study did not meet its primary objective of a superiority trial aimed at demonstrating a 0.6 cm difference in favor of PRP on the VAS scale. There was no significant change in the disability scores. Subgroup analyses were performed to identify the treatment response by patient characteristics such as pain intensity at baseline and skin type. As expected, safety data showed erythema and skin discoloration in the PRP group but not in the control group.

**Discussion/conclusion:**

This trial had multiple limitations that need to be addressed in future research. Although the primary objective was not achieved, this proof-of-concept study provides important efficacy and safety data in relation to the use of blue light in the treatment of chronic back pain and key insights that may support further research on similar devices.

**Clinical Trial Registration:**

ClinicalTrials.gov, identifier NCT01528332.

## 1 Introduction

The global prevalence of low back and neck pain is one of the highest and contributes significantly to physical disability with the consequent socio-economic burden (1, 2). The World Health Organization (WHO) estimated that in 2020, 619 million individuals globally experienced low back pain, with a projection of 834 million cases by 2050 (WHO 2023). One in seven primary care consultations address musculoskeletal concerns, with back pain being the most common (3).

Currently, there is no universally accepted standard for the treatment of low back and neck pain despite its widespread occurrence and the resulting adverse socio-economic impact. The majority of European guidelines recommend non-pharmacological intervention as the primary approach for managing back pain, such as physiotherapy and cognitive behavioral therapy (4). However, despite these recommendations, non-pharmacological treatments face challenges in gaining widespread use by patients and clinicians due to low acceptance, low adherence (5), access and cost issues, and so on (6).

For the above reasons, and despite their long-term modest to no efficacy and recommended short-term use due to side effects (IASP Fact Sheet), pharmacological interventions remain the treatment of choice in chronic back pain (7). The opioid crisis, particularly prevalent in the United States (8), underscores the limitations of this approach, with the potential for abuse, dependence, misuse, and tolerance often outweighing the short-term benefit. Similarly, the risk of abuse with other systemic treatments such as benzodiazepines (9, 10) and gabepentinoids (11) highlights significant unmet needs in the treatment of chronic back pain.

Clearly, a treatment that is efficacious, safe, affordable, and conducive to long-term adherence is still needed for the treatment of back pain. Blue light (BL) phototherapy has been used for the treatment of acne vulgaris, psoriasis (12), and neonatal jaundice (13) and shows potential for pain relief (14). Upon skin exposure to blue light, optovin transiently binds to TRPA1 through cysteine residues (15). This phenomenon has sparked the idea that blue light may act as a TRPA1 antagonist and could therefore be used to modulate pain. In healthy volunteers, 1 h exposure to BL substantially reduced spontaneous pain as assessed by Numeric Rating Scale (NRS) and altered the quality of pain (14). BL may therefore reduce spontaneous and evoked pain by modulating activated peripheral nociceptive fibers in human skin (14).

**Figure 1:**
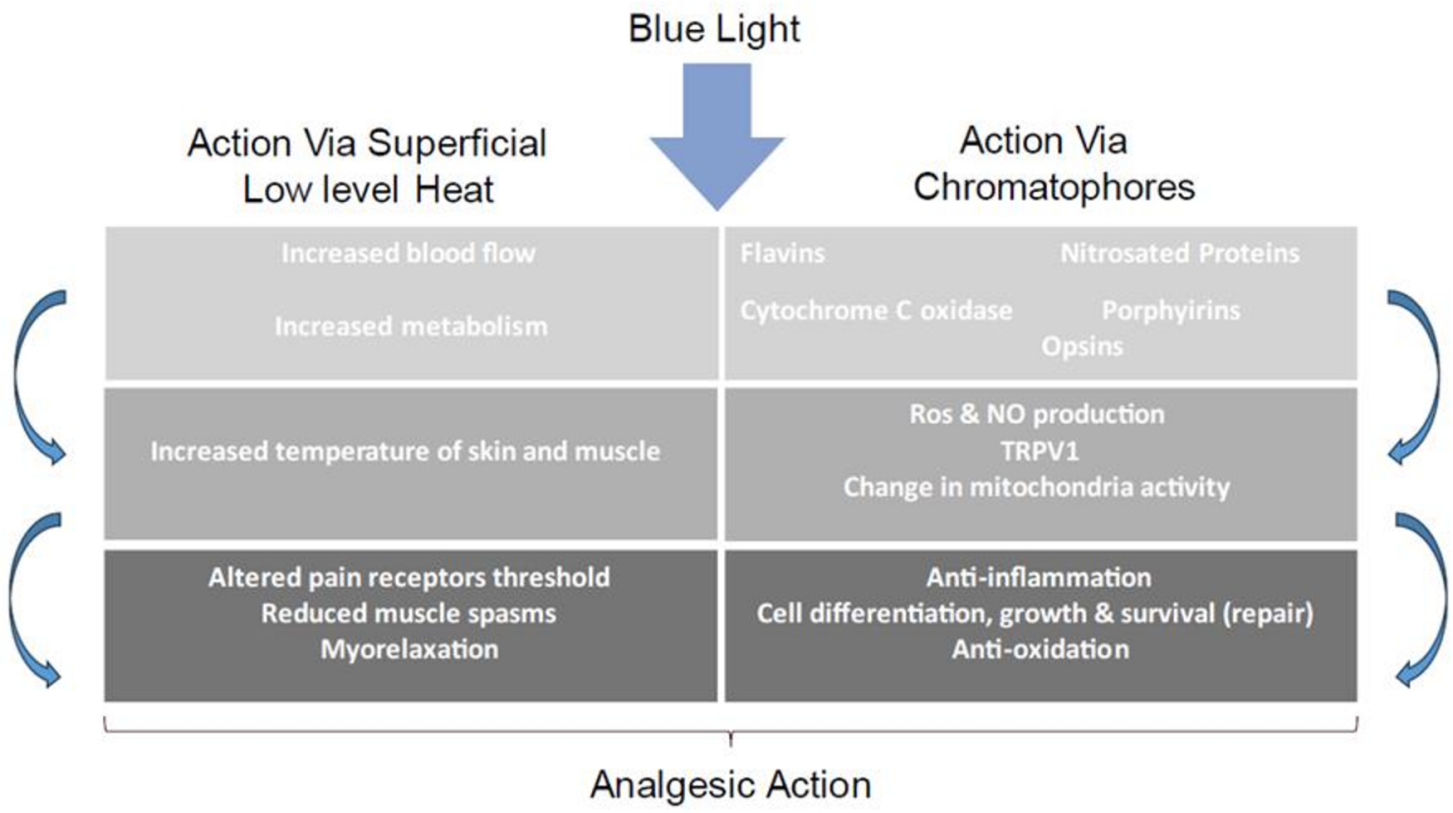
Proposed mechanism of action of blue light phototherapy.

The reduction in pain and inflammation by blue light is thought to occur through a complex cascade of events (12, 14, 16) (Figure 1).

**Figure 2:**
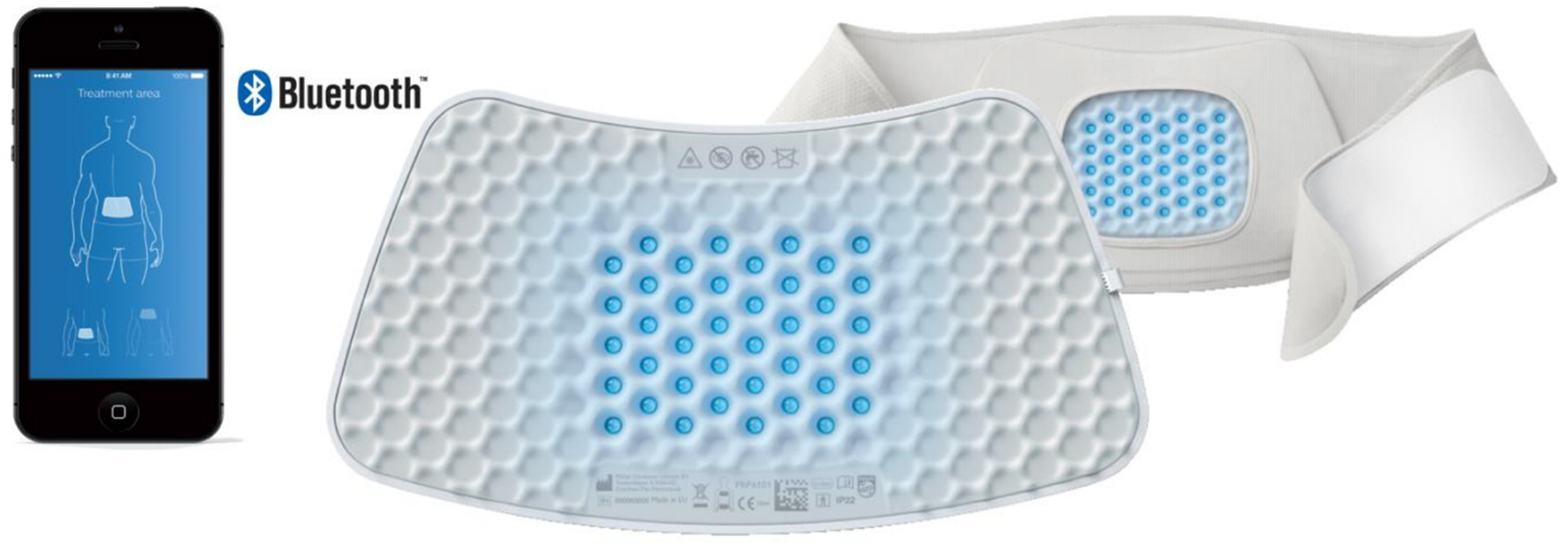
Pain relief patch, viewed from the rear of the lower back strap. The user interface and connection are by means of a smartphone.

This study explores the efficacy and safety of a non-invasive medical device, the pain relief patch (PRP), in the treatment of musculoskeletal back pain. The device delivers blue light phototherapy and heat directly to the affected area through optical power and thermal conduction. It comprises a fabric panel of 40 integrated blue LEDs with a peak wavelength of 453 ± 7 nm at an average power density of 20 ± 1 mW/cm^2^. The user interface is controlled by an app on a smartphone (Figure 2). In normal use, the PRP was designed not to exceed a temperature of 43°C (in line with applicable European norms). During treatment, the PRP is placed in the pocket of the accompanying fabric belt (for the lower back) or harness (for the upper back).

The study, designed as a proof-of-concept, prospective, randomized, double-blind, controlled, parallel-group clinical trial, aimed to investigate the superiority of PRP vs. a control device in the reduction of mild to moderate chronic musculoskeletal back pain after five treatment sessions.

## 2 Materials and methods

This clinical trial was registered under number NCT01528332 and lasted from 3 February 2012 to 3 July 2012. It was undertaken by Philips Electronics N.V. (Eindhoven, Netherlands), the company that originally commercialized the PRP device, but the data were never published. PRP is now licensed to Heat2Move B.V. (H2M), a company working on an improved version of the PRP, which has full publication rights to the data. H2M had access to all study data and documents, including but not limited to the study protocol, statistical analysis plan, statistical analyses, anonymized aggregated data, and the clinical study report. All statistical analyses and results were already available and performed in a masked way by Philips. However, the individual non-anonymized raw data were not accessible to H2M as the informed consent did not foresee any data transfer to a third party other than the Contract Research Organization (CRO).

The study was designed as a proof-of-concept, comparative efficacy and safety trial and was undertaken across three different clinical sites at the University Hospitals of Heidelberg and Mannheim, Germany. The CONSORT guidelines for RCT reporting were followed.

This superiority study aimed to compare five treatment sessions of 30 min each with blue light (PRP) vs. five treatment sessions of 5 s with green light (control device).

### 2.1 Participants

Following a screening visit at which informed consent was obtained, participants were included in the study if they fulfilled the criteria summarized in Table 1.

**Table 1:** Main medical inclusion and exclusion criteria.

Inclusion criteria	• 18–65 years old • Skin type II, III, or IV on the Fitzpatrick skin type scale • Able to consent to participation by signing the informed consent form • Back pain localized in the upper (shoulders level) or lower back • Pain present for at least 3 months and with a mild to moderate intensity (score between ≥2 and ≤6 on a 0–10 point on pain intensity VAS) prior to enrollment in the study
Exclusion criteria	Participation in another clinical study at the time of this study or within the 30 days prior to signing the informed consent Moderate to severe arterial hypertension History of stroke or myocardial infarction Peripheral vascular disease or severe congestive heart failure Poor general health Failed back surgery or surgery to the torso, head, or back within the previous 8 weeks Acute dislocation or fracture within the previous 8 weeks Degenerative central nervous system disease Spinal stenosis associated with pain or cauda equina syndrome Neurological symptoms indicating neuropathy or widespread pain Inflammatory diseases that cause pain or any other chronic disease or infection known to cause pain Severe depression Cancer Severe osteoporosis and another severe bone disease Unwillingness to abstain from other non-drug back pain treatments during the trial Unwillingness to abstain from the use of pain medication other than those recommended in steps 1 and 2 of the WHO analgesic ladder for the duration of the trial Known sun allergy, use of photosensitizing medication, or diseases that cause photosensitivity Active implantable medical devices such as cardiac pacemakers Pregnant or breast-feeding women and sexually active females of childbearing potential not using a medically approved form of contraception

As a precautionary approach, the exclusion criteria were extensive as data on phototherapy side effects were limited at the time of execution of the study, particularly in the chronic pain population.

### 2.2 Randomization, investigator masking, and study schedule

Allocation to the control or PRP treatment device was undertaken immediately prior to the first treatment (visit 1) and was based on a 1:1 block randomization stratified by site. It was administered by site staff, who were masked to the device, using the Randomizer, an independent audit-trailed web-based system (Institute for Medical Information). The Randomizer was also used for unmasking individual participants in the event of any side effects. The control device was identical in appearance to the PRP but consisted of green LEDs with a wavelength of 531 ± 7 nm. In normal use, the control device reaches a maximal temperature of 36–37°C. To maintain masking of the participants to the treatment, patients were not made aware of the temperature difference between the active and the control device. The instructions for use simply stipulated that some heat may be felt during the treatment session and that the individual’s skin type might interfere with the temperature felt.

The allocation schedule and the list of the serial numbers of the corresponding device were kept in locked facilities by the CRO, which was responsible for the implementation and execution of the study.

**Figure 3:**
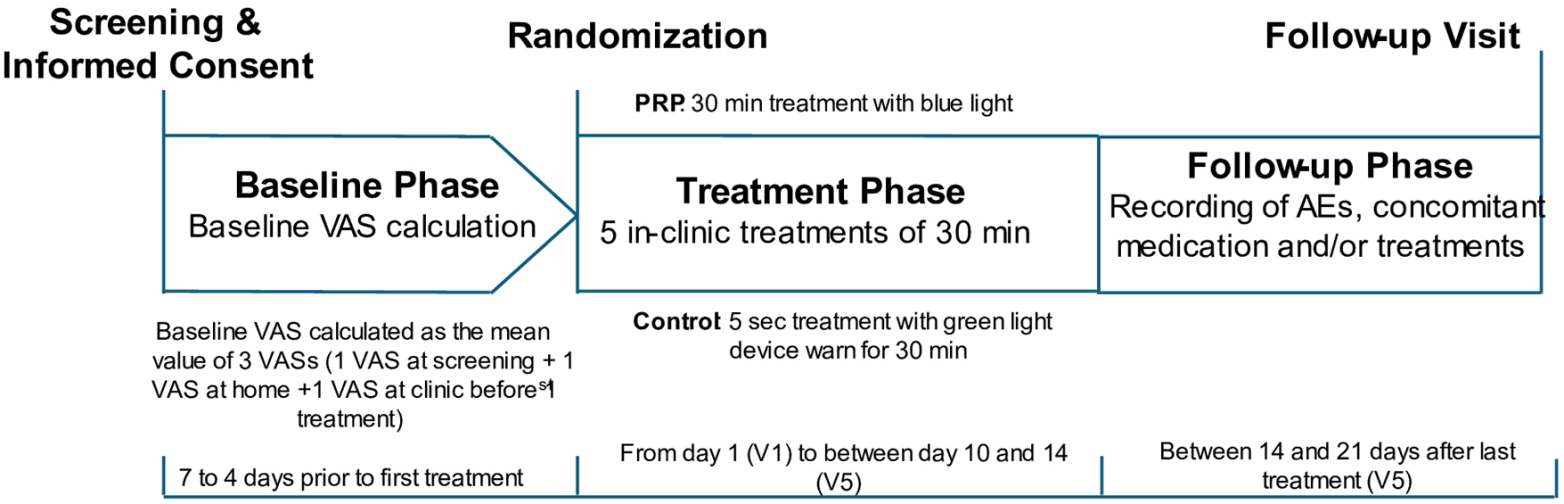
Schematic illustrating the study design: both control and PRP groups wore the device for 30 min. The control device was turned off automatically 5 s after activation, while the PRP stayed on for the full 30 min.

The participants attended five treatment sessions over a period of 10–14 days (Figure 3). At each treatment visit, participants completed pre- and post-treatment assessments, including pain intensity, vital signs, and measurements of skin erythema and pigmentation levels in the treatment area. The latter were measured objectively with the Mexameter (MX-18; CK Electronic, Cologne, Germany), which uses principles of light absorption and reflection.

Two to three weeks after the final treatment visit, participants returned to the clinic for a follow-up visit.

All activities that could lead to inadvertent unmasking of the trial were performed by unmasked study personnel. This included positioning and removing the device, turning it on, verifying that it operated correctly, conducting device maintenance, and assessing post-treatment skin conditions.

Each treatment session was under the constant supervision of a masked study team member. Participants and masked study team personnel wore goggles that blocked blue and green light to prevent any unmasking of the treatment allocation. Participants were allowed to lie down (in a position avoiding any pressure on the device), stand, or walk.

Pain intensity was calculated at baseline and scored by each participant before and after each treatment session using an 11-point visual analog scale (VAS), with the endpoints 0 equating to “no pain” and 10 equating to “worst pain imaginable.” The effect of pain on daily activities was measured by the Roland–Morris Disability Questionnaire (RMDQ), a 24-point scale, prior to treatment at visit 1, following treatment at visits 3 and 5, and at the follow-up visit.

The Ethics Committee of the University Clinic, Heidelberg served as the study ethics committee.

### 2.3 Outcomes

The primary objective of the study was to demonstrate the superiority of the treatment with the PRP vs. the control device by comparing the changes in the VAS pain intensity score from baseline to post-treatment. The baseline VAS pain intensity score was calculated as the mean of three VAS scores (at the screening visit, at home, and at the first visit immediately prior to the first treatment). The post-treatment VAS score was calculated as the mean of the post-treatment scores obtained across the five treatment visits. The choice to use the aggregate post-treatment mean VAS scores across the five treatment sessions was justified by the fact that no data were available in relation to the most efficacious number of treatment sessions. The possible fluctuation of the treatment response across sessions was mitigated using this approach.

Planned subgroup analyses were performed based on pain location (upper/lower back), sex, baseline pain intensity (VAS pain intensity <4 vs. ≥4 in an attempt to analyze separately patients with mild vs. moderate pain at inclusion), skin type (Fitzpatrick skin types II/III/IV), and body mass index (<18.5, 18.5–25, 25–30 and >30).

Secondary objectives included safety, comparison of the change vs. baseline of the RMDQ scores in the control and PRP groups at the final treatment session (visit 5), and the differences between the treatment groups in the VAS pain intensity score at the follow-up visit.

### 2.4 Sample size and statistical analysis

The magnitude of the difference in the VAS score between treatment and comparator groups, which is considered clinically meaningful, is equivocal (17). In the current study, the choice of a between-group difference in the change from baseline in the VAS score of 0.6 is debatable, as it is very small and was not justified nor documented in the clinical study protocol, other than by the fact that the study is a proof of concept and a signal needed to be detected.

All participants who were treated at least once were included in the “full analysis set” (FAS; intention-to-treat population). All participants with no major protocol deviations were included in the “per protocol set” (PPS). The FAS was the primary population for all efficacy and safety analyses.

The treatment effects and the treatment difference between the control and PRP groups were estimated by least squares (as point estimates) and the corresponding two-sided 95% confidence interval (CI) computed based on the *t*-distribution. Two sensitivity analyses were performed on the FAS, where the average VAS pain intensity was derived by imputing missing values of post-treatment VAS pain intensity ratings using two methods. Method 1 used the worst post-treatment value carried over backward/forward, and Method 2 used the worst post-treatment VAS value available for the PRP group and the best VAS value available for the control group. The changes in VAS and RMDQ scores at the Follow-up compared to the last treatment (visit 5) were descriptively analyzed within each treatment group with a 95% confidence interval. No between-group comparisons were made.

Safety data were analyzed from the FAS by descriptive statistical methods. As a rule, if an adverse event was missing the intensity, seriousness, or causality assessment, it was substituted with the worst-case outcome.

## 3 Results

Based on the assumption of a standard deviation (SD) of 1.2 in the between-group difference in the VAS score, 73 patients per group were required to detect a difference of 0.6 with a power of 85% and a type I error of 5%. A sample size of 170 participants (85 patients in each group) was required to allow for a drop-out rate of 14%.

**Figure 4:**
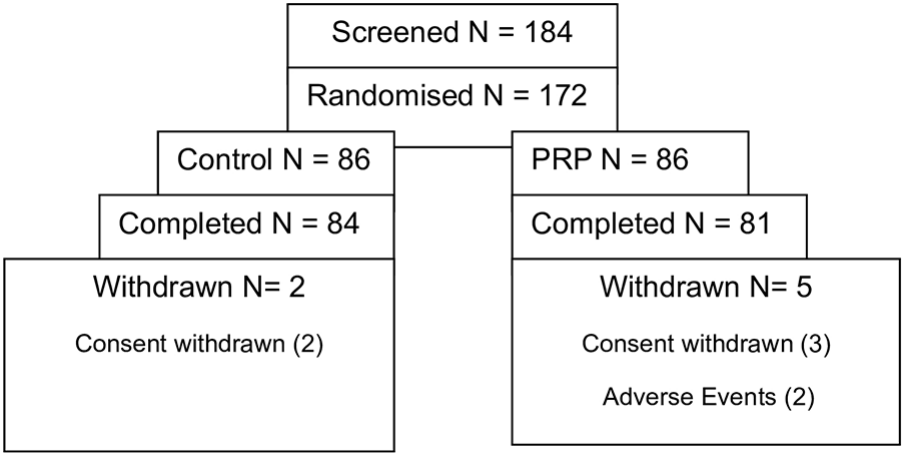
Patients’ disposition by the treatment group.

The FAS included 171 patients who received at least one treatment session, and the PPS included 157 patients who were treated without any protocol violation. Safety analysis was based on the FAS (Figure 4).

No differences in demographic data were observed between the control and PRP groups (Table 2). In total, 99.4% of patients were Caucasian and with a Type III Fitzpatrick Skin type (medium white to olive) (control 70.9%; PRP 72.9%). At baseline, the RMDQ score of the control group was 0.8 points greater than that of the PRP group.

**Table 2:** Demographic and baseline characteristics—full analysis set.

	Control (*N* = 86)	PRP (*N* = 85)	Total (*N* = 171)
	*n* (%) or mean + SD	*n* (%) or mean ± SD	*n* (%) or mean ± SD
Gender			
Male	28 (32.6)	30 (35.3)	58 (33.9)
Female	58 (67.4)	55 (64.7)	113 (66.1)
Ethnic group			
Caucasian	85 (98.8)	85 (100.0)	170 (99.4)
Other	1 (1.2)	0 (0.0)	1 (0.6)
Age (years)			
Mean ± SD	51.4 ± 8.3	50.0 ± 10.7	50.7 ± 9.5
Min/Median/Max	24/52/65	22/52/65	22/52/65
BMI (kg/m^2^)	26.10 ± 4.09	26.55 ± 5.27	26.32 ± 4.71
Skin type			
Type II	10 (11.6)	9 (10.6)	19 (11.1)
Type III	61 (70.9)	62 (72.9)	123 (71.9)
Type IV	15 (17.4)	14 (16.5)	29 (17.0)
RMDQ	6.6 ± 4.2	5.8 ± 3.7	6.2 ± 3.95

The pain characteristics at enrollment were similar between the two groups, with most participants (73.3% in control and 72.9 in PRP) suffering from low back pain, and the median time to onset of the initial pain was 61.6 months in the control group and 60.2 months in the PRP group. In total, more than half of the FAS participants (61.6% in the control and 51.8% in the PRP group) had suffered from back pain for more than 5 years (Table 3).

**Table 3:** Baseline pain characteristics in the full analysis set by the location and duration of back pain.

	Control (*N* = 86)	PRP (*N* = 85)	Total (*N* = 171)
	*n* (%)	*n* (%)	*n* (%)
Location of back pain			
Lower back	63 (73.3)	62 (72.9)	125 (73.1)
Upper back	23 (26.7)	23 (27.1)	46 (26.9)
Time since first onset			
Median	61.56	60.16	60.72
Min/Max	1.5/531.0	5.1/422.6	1.5/531.0
Up to 1 year	6 (7.0)	7 (8.2)	13 (7.6)
>1–2 years	2 (2.3)	8 (9.4)	10 (5.8)
>2–5 years	23 (26.7)	25 (29.4)	48 (28.1)
>5 years	53 (61.6)	44 (51.8)	97 (56.7)
No data (unknown)	2 (2.3)	1 (1.2)	3 (1.8)

Prior pain treatments were defined as those used during the immediate 3 months before inclusion in the study and discontinued before the first treatment (visit 1). Overall, 165 participants (96.5% of all FAS participants) had received at least one treatment for back pain prior to entering the study. Physiotherapy (81.9%), medication (78.4%), and therapies such as massages, baths, cryo/heat therapies (69.0%), and injections in the painful area (43.3%) were the most frequent treatments in both study groups. Other less frequent treatments included acupuncture (39.8%), transcutaneous electrical nerve stimulation (31.0%), rehabilitation treatment (30.4%), and relaxation techniques (25.1%).

Anti-inflammatory drugs, as concomitant pain medication, were given to almost half the participants within the full analysis set (46.8%), while analgesics were given to 30.4%. The use of analgesics had not been analyzed by class; therefore, no data were available on step 1 and 2 treatments of the WHO pain management ladder. Consequently, the use of the so-called “weak opioids,” such as tramadol, was not documented in the study.

**Figure 5:**
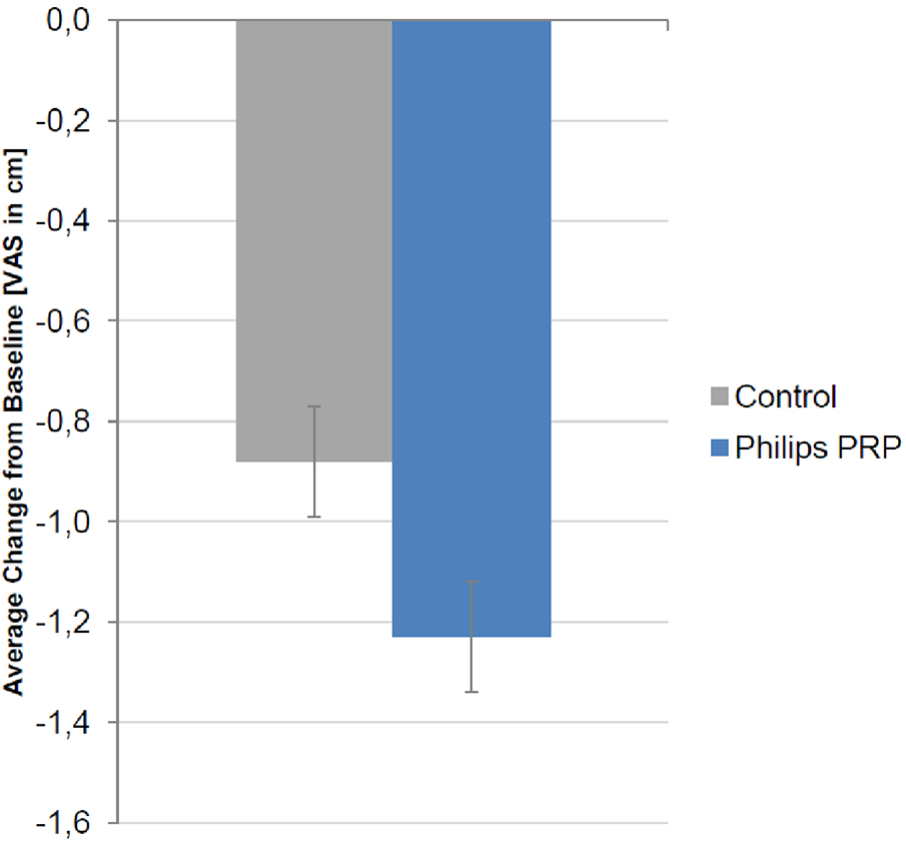
Change from baseline in the mean VAS pain intensity score (cm) over the five treatment visits for the full analysis set. A negative value signifies a reduction in pain intensity. The reduction was −1.24 ± 0.11 cm in the PRP group and −0.87 ± 0.11 cm in the control group. The treatment difference was −0.37 (95% CI, −0.67 to −0.06) (*p* = 0.0196, *F*-test, ANCOVA).

The primary outcome, the change from baseline in the mean VAS pain intensity score across the five treatments for the control and the PRP devices, is summarized in Figure 5.

A decrease in the VAS pain intensity was present in the FAS for both treatment groups, with the improvement being more pronounced in the PRP group. The difference in treatment between the control and PRP groups of −0.37 cm (95% CI −0.67 to −0.06) reached statistical significance (*p* = 0.0196, *F*-test, ANCOVA) but did not confirm the study superiority hypothesis of a difference between the PRP and the control groups of 0.6 cm on the VAS. Two sensitivity analyses were performed using the two different imputation methods for missing values; both methods confirmed the results of the FAS primary analysis (*p* < 0.05, two-sided).

The difference in treatment in the PPS was −0.38 cm (95% CI, −0.70 to −0.06 cm) (*p* = 0.0221, *F*-test, ANCOVA).

**Figure 6:**
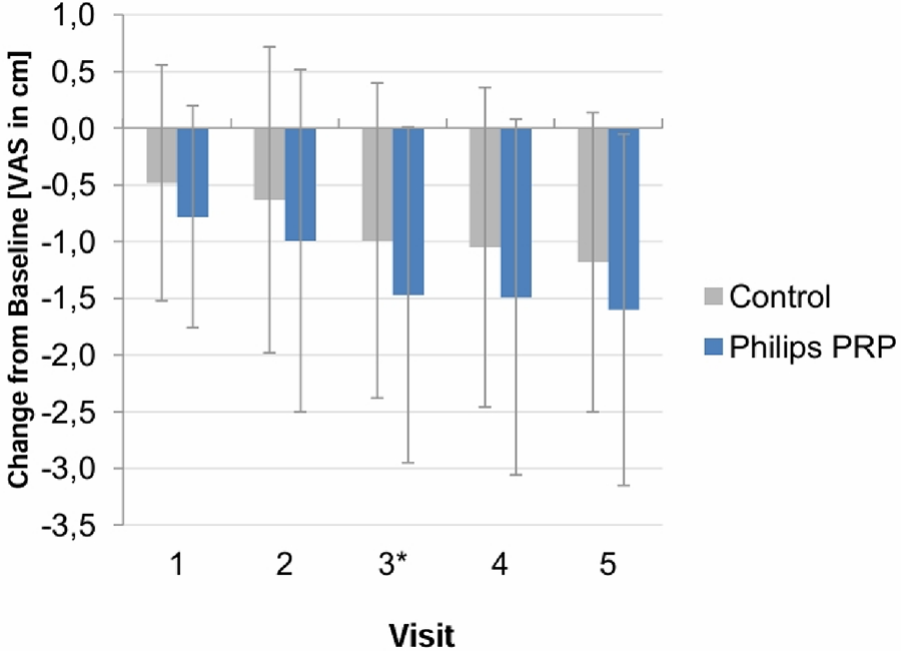
Post-treatment VAS pain intensity score (mean ± SD) by visit—full analysis set. Vertical lines indicate mean ± SD.

The analysis of post-treatment pain intensity by visit is shown in Figure 6.

The VAS pain intensity values did not appear to be constant over time. Additional descriptive statistics were conducted to determine whether, and at what time point, differences between the treatment groups reached statistical significance. Significant treatment differences were found at visit 3 post-treatment and for the average post-treatment difference (average of post-treatment values of visits 1–3). A trend favoring PRP was observed for visits 1, 2, 4, and 5, but it did not reach statistical significance.

The treatment efficacy by subgroup is summarized in Table 4.

**Table 4:** Change in mean (SD) VAS pain intensity score (cm) from baseline to visit 5 by subgroup in the full analysis set.

	Control (*N* = 86)	PRP (*N* = 85)
	*n* (mean ± SD)	*n* (mean ± SD)
Baseline VAS pain intensity (cm)		
<4 cm	44 (−0.70 ± 0.87)	44 (−0.88 ± 0.78)
>4 cm	42 (−1.07 ± 1.11)	41 (−1.62 ± 1.29)
Sex		
Male	28 (−1.01 ± 0.88)	30 (−0.94 ± 0.85)
Female	58 (−0.82 ± 1.06)	55 (−1.39 ± 1.21)
Skin type (Fitzpatrick)		
Type II	10 (−1.05 ± 1.24)	9 (−1.24 ± 0.72)
Type III	61 (−0.99 ± 1.00)	62 (−1.22 ± 1.14)
Type IV	15 (−0.32 ± 0.71)	14 (−1.29 ± 1.24)
Location of back pain		
Lower back	63 (−0.98 ± 1.06)	62 (−1.10 ± 1.12)
Upper back	23 (−0.61 ± 0.81)	23 (−1.60 ± 1.02)
Body mass index (kg/m^2^)^a^		
18.5 to <25.0	39 (−0.72 ± 1.11)	35 (−1.10 ± 1.02)
25.0 to <30.0	26 (−0.98 ± 0.72)	31 (−1.30 ± 1.13)
≥30.0	21 (−1.06 ± 1.10)	18 (−1.39 ± 1.30)

^a^ The 18.5-kg/m^2^ body mass index category is not displayed (it contained only one participant from the PRP group).

All subgroup analyses, except by difference in sex, favored treatment with the PRP over the control device, although all differences were mostly modest. The subgroup analysis by intensity of pain at baseline showed a higher control and PRP efficacy in participants with a baseline pain intensity of 4 cm or more. The subgroup analysis by location of pain showed a mean (SD) change from baseline in VAS pain intensity for the upper back subgroup of −1.60 cm (SD 1.02) for the PRP group compared to that of −0.61 cm (SD 0.81) for the control group. An ANCOVA performed *post hoc* on the FAS with baseline pain intensity as a covariate found a treatment difference between PRP and control in average VAS pain intensity from baseline of −0.97 cm with a *p*-value of 0.001. In the lower back subgroup, the mean change from baseline for the PRP group was −1.10 cm (SD 1.12) compared to −0.98 cm (SD 1.02) for the control group. This between-group treatment difference was not statistically significant (*p* < 0.440).

The subgroup analysis by skin type favored the PRP for all skin types. Participants with skin type IV manifested the highest treatment differences [−0.32 cm (SD 0.71) in the control group compared to −1.29 cm (SD 1.24) in the PRP group].

In the PRP group, participants with a higher body mass index (BMI) (30.0 kg/m^2^) had a better response than those with a lower BMI, although, due to the smaller number of participants in this subgroup and the higher SD, this trend must be interpreted with caution.

The secondary efficacy endpoint change, the difference between the VAS pain intensity score at the last treatment and that at the follow-up visit, is summarized in Table 5.

**Table 5:** Difference in the VAS pain intensity score at the last treatment and at the follow-up visit for the full analysis set.

Parameter	Control (*N* = 86)	PRP (*N* = 85)
	*n* (mean ± SD)	*n* (mean ± SD)
VAS pain intensity (cm)		
Last treatment	86 (2.79 ± 1.68)	85 (2.34 ± 1.64)
Follow-up	85 (2.83 ± 1.80)	83 (2.59 ± 1.59)
Change from treatment	85 (0.04 ± 1.59)	83 (0.24 ± 1.68)
95% CI for mean change	−0.31 to 0.38	−0.13 to 0.60

The VAS pain intensity score sensibly increased (i.e., worsened) in each treatment group when treatment was stopped [control: 0.04 (SD 1.59), PRP: 0.24 (SD 1.68)]. Similar results were observed for the PPS.

The secondary endpoint, “functionality,” was measured using the RMDQ. The mean values of the RMDQ scores across the study for the FAS are given in Table 6.

**Table 6:** RMDQ during the study course for the full analysis Set.

Visit	Control (*N* = 86)	PRP (*N* = 85)
	*n* (mean ± SD)	*n* (mean ± SD)
Baseline (treatment visit 1)	85 (6.6 ± 4.2)	85 (5.8 ± 3.7)
Treatment visit 3	85 (5.8 ± 4.5)	81 (5.5 ± 3.9)
Treatment visit 5	84 (5.6 ± 4.0)	81 (5.0 ± 4.1)
Follow-up visit	83 (5.3 ± 4.4)	81 (4.8 ± 4.0)

The RMDQ decreased across the visits in both treatment groups. It was lower for the PRP group than in the control group, but the difference did not reach statistical significance. The PPS confirmed the results obtained for the FAS.

The percentage of treatment compliance was comparable between the treatment and control groups and ranged from 100% to 106.7%, indicating an overuse of the devices by a few patients. Compliance of less than 80% was observed in six patients (two in the control group and four in the PRP group) who prematurely terminated the study.

Serious adverse events (SAEs) and adverse events (AEs) were recorded from the screening visit to the follow-up visit. No SAEs were seen during the study. A total of 104 participants (60.8%) experienced treatment emergent adverse events (TEAEs): 53 (61.6%) in the control group and 51 (60.0%) in the PRP group. Table 7 presents an overview of the AEs.

**Table 7:** Overview of adverse events.

	Control (*N* = 86)				PRP (*N* = 85)				Total 9 (*N* = 171)			
	*n*	%	AE	Rate	*n*	%	AE	Rate	*n*	%	AE	Rate
SAE	0	0	0	0	0	0	0	0	0	0	0	0
TEAE												
Total	53	61.1	195	2.27	51	60.0	185	2.18	104	60.8	380	2.22
IMD related	11	12.8	20	0.23	11	12.9	20	0.24	22	12.9	400	0.23
Procedure-related	9	10.5	16	0.19	5	5.9	14	0.16	14	8.2	30	0.18
Leading to discontinuation	1	1.2	1	0.01	22	2.4	2	0.02	3	1.8	3	0.02

*n*, number of patients with at least one AE: individual patients may have reported more than one AE; Rate, incidence rate of individual adverse events; IMD, investigational medical device.

No meaningful differences were observed in the distribution of TEAEs between the two treatment groups except for erythema and skin discoloration. The majority of TEAEs in both groups were mild and required no intervention. Headache was the most common AE and was present in both groups. During the treatment and follow-up phases, 30 patients (34.9%) in the control group reported a total of 72 headaches, while 27 patients (31.8%) in the PRP group reported a total of 61 headaches.

The blue light (PRP) treatment was expected to induce transient erythema and temporary increases in skin melanin levels, leading to skin discoloration (tanning) in the treatment area. Melanin values increased from baseline in the PRP group (fluctuating between 3.8 and 14.4 Mexameter units) but not in the control group. No differences were detected between the control and PRP groups in vital signs at any time.

## 4 Discussion

This proof-of-concept controlled trial investigated the efficacy and safety of blue light phototherapy in back pain. A total of 171 patients with chronic mild to moderate musculoskeletal back pain were treated over 14 days by the PRP or a control device, which emitted a green light.

To our knowledge, this is the first sizable randomized controlled clinical trial using blue light for treating chronic back pain. The PRP and its characteristics underwent extensive technical validation and received Conformité Européenne (CE) marking. The study data, including subgroup analyses, explored the response by patients and pain characteristics.

The study design had a few limitations such as the lack of a washout period from analgesics before inclusion in the study and the multitude of covariates (location of pain, intensity of pain at baseline, skin types, etc.) that could have influenced treatment outcomes. In addition, the extensive exclusion criteria used in the study narrowed extensively the patient profiles studied in this trial. The inclusion of patients with mild pain complicates to analysis of the results, as demonstrating a clinically significant decrease in pain intensity in this population is challenging due to the low pain scores at baseline. Including patients with moderate to severe pain would have been preferable. Furthermore, the study did not use any tools to identify the etiology of pain, which makes it difficult to assess the efficacy of blue light phototherapy by pain etiology.

After five treatments, the VAS pain intensity score in both treatment groups improved from baseline, but the improvement was significantly greater for the PRP group (*p* < 0.02), although the difference did not confirm the superiority hypothesis. Pain assessment using the VAS score can only indicate the magnitude of a decrease or increase in pain intensity. It is less useful in assessing the success of a treatment as defined by patients. Nevertheless, it is suggested that a reduction of 3.0 cm represents a clinically important difference corresponding to the patients’ perception of adequate pain control (18). The VAS decrease in the PRP group of −1.24 cm (SD 0.11), although at best modest and below the hypothesized 0.6 cm, provides a signal that needs to be confirmed in a study with better methodology (e.g., inclusion and exclusion criteria, primary objective, etc.). An analysis based on the number of participants with a 30% and 50% reduction in their pain after treatment would have been a better approach; however, as we could not obtain the raw data from Philips, such an analysis could not be performed.

The outcomes of pain trials are also notoriously difficult to delineate because of the high placebo effect (19), and this may have contributed to reducing the difference observed between the study groups. In addition, a dose curve available before this trial meant that the duration of a treatment session and the number of sessions required for an optimal outcome were derived from published data and the hypothesized mechanism of action. To mitigate this risk, the primary outcome used an aggregate value from the five treatment sessions, but the detailed response by session showed that the difference between the PRP and the control device was only statistically significant in session 3. A more detailed dose curve, including the response by intensity, treatment session duration, and the number of treatment sessions, would have helped identify the optimal treatment duration and a primary outcome based on comparing the pain intensity at the last treatment vs. baseline.

The use of green light may also have affected the observed differences since green light has demonstrated effective in pain management (20). In addition, participants were allowed to continue their step 2 WHO analgesic ladder treatments, including opioids. The data on the stratification of the presence or absence of opioids during the trial were not available. Therefore, it was not possible to determine the efficacy of the PRP by the presence or absence of opioid use. It is important to highlight that the control group was “more disabled” than the PRP group by a difference of 0.8 points. This may explain the absence of a significant difference in the RMDQ results between the two groups, knowing that a higher RMDQ score may be more sensitive to an intervention than a lower one (21). One may assume that the higher RMDQ score in the control arm would translate into more patients with higher pain intensity (>4) in this group; however, as shown in Table 4, the number of patients with pain intensity below 4 or equal to and higher than 4 was similar in the PRP and the control groups.

In addition, the duration of the study may not have been long enough to capture changes in the functionality of participants. The low RMDQ scores in both groups indicate that the study included participants who may not have had disability scores to easily detect improvement by RMDQ within the given sample size and study duration. It is also important to note that subgroup analyses indicated that the PRP was more efficacious for skin type VI, although this group was a minority in the study.

The change in VAS pain intensity from the last treatment visit to the follow-up visit showed a slight increase in each treatment group. This increase was more pronounced in the PRP group compared to that in the control group. Given that pain reduction was greater with PRP, it is expected that discontinuing its use could bring pain to its baseline values, which may explain the more rapid increase observed in the PRP group compared that in the control group.

No substantial differences in safety outcomes were seen between groups treated with the PRP and the control device. As expected, transient erythema and skin discoloration were reported in the PRP group and not in the control group, highlighting the need to inform patients who may use this device of this change and explain its transient nature in most patients. The effects of long-term use of blue light are not documented and need further investigation. Blue light may interact with the circadian rhythm and damage the eyes if used inappropriately, necessitating the importance of correct testing and instruction of use of any device using blue light.

A new study using an improved device combining blue and infrared red light is under preparation to substantiate these findings using the information on optimal delineated in this study to improve the study design.

In conclusion, although it did not demonstrate the superiority of the PRP over the active control device, this study identified a signal from an efficacy point of view (albeit too small to be clinically relevant), which warrants further investigation with a better study design.

## Data Availability

The raw and anonymised data supporting the conclusions of this article will be made available by the authors without undue reservation.
